# Green and Rapid Determination of Phthalates in Mineral Water From Virgin and Recycled PET Bottles

**DOI:** 10.1002/jms.70089

**Published:** 2026-07-16

**Authors:** Claudia Lino, Serena Indelicato, David Bongiorno, Fabio D'Agostino, Giuseppe Avellone

**Affiliations:** ^1^ Department of Biological, Chemical and Pharmaceutical Science and Technology (STEBICEF) University of Palermo Palermo Italy; ^2^ Institute of Anthropic Impacts and Sustainability in the Marine Environment (IAS) National Research Council of Italy (IAS‐CNR) Trapani Italy

**Keywords:** PET bottles, phthalates, SPME‐GC/MS, water

## Abstract

This study investigates the presence and quantification of phthalates released into mineral waters from virgin and recycled polyethylene terephthalate (PET) bottles. A green, solvent‐free analytical method based on solid‐phase microextraction (SPME) coupled with gas chromatography–mass spectrometry (GC/MS) was developed and validated to detect the following four target phthalates: diethyl phthalate (DEP), diisobutyl phthalate (DiBP), dibutyl phthalate (DBP), and bis(2‐ethylhexyl) phthalate (DEHP). The analytical method showed good sensitivity, precision, accuracy, and low limits of detection and quantification. Seventeen commercial water samples were analyzed, including 11 from virgin PET and six from recycled PET bottles with varying recycling content (30%–100%). Phthalate concentrations ranged from 2.21 to 18.96 μg/L, with DEHP being the most prevalent compound. Risk assessment following EFSA guidelines demonstrated that the estimated daily intakes (DIs) and the risk quotient (RQ) of all detected phthalates were well below the tolerable DI limits for both adults and toddlers. These findings confirm that the proposed method is suitable for routine screening of phthalates in mineral water and highlight the need for stricter quality controls in the recycled PET supply chain.

## 1 Introduction

Phthalates (PAEs), a class of synthetic organic compounds commonly used as plasticizers, are among the most prevalent classes of endocrine‐disrupting chemicals (EDCs) in consumer products. Because they are not covalently bound to polymer matrices, they can readily migrate into the surrounding environment, including air, food, and water [1, 2, 3].

Growing evidence indicates that their endocrine‐disrupting properties are associated with a range of adverse health effects, particularly involving reproductive, developmental, and metabolic functions [4, 5, 6, 7]. Despite these risks, several phthalates remain unregulated under EU water quality directives, although the EU recognizes many as endocrine disruptors [8, 9].

In recent years, bottled water has emerged as a potentially significant route of human phthalate exposure. Italy, for example, leads Europe in bottled water consumption, with an average of 252 L per capita per year, far exceeding the EU average of 88 L [10].

Given the widespread use of polyethylene terephthalate (PET), both virgin and recycled, for water packaging, concerns have arisen regarding phthalate migration during storage, especially under elevated temperatures or prolonged contact. Regulation (EU) No. 10/2011 governs plastic materials and articles intended to come into contact with foodstuffs. Within this regulation, certain phthalates e.g., dibutyl phthalate (DBP), benzyl butyl phthalate (BBP), di(2‐ethylhexyl) phthalate (DEHP), diisononyl phthalate (DINP), and diisodecyl phthalate (DIDP) may be authorized; however, their use is subject to specific restrictions and limited to certain applications [11].

PET is a polyester synthesized from terephthalic acid and ethylene glycol, prized for its strength, transparency, and chemical resistance; importantly, PET does not contain phthalates; they are not used in its polymerization or processing. Phthalates are likely added primarily to provide flexibility to polymers, or may come from external sources, such as environmental or process contamination [12]. In response to increasing environmental concerns and the need to reduce plastic waste, the development and widespread use of recycled PET (rPET) bottles have been actively promoted in recent years. While rPET contributes to a more circular economy, it also introduces additional challenges related to the potential presence of legacy contaminants, including phthalates. Therefore, careful control of feedstock quality, purification technologies, and compliance with food‐grade safety standards is essential to minimize the migration of undesired compounds into bottled water. However, traces of phthalates have occasionally been detected in water stored in PET bottles, which is a cause for concern [13, 14, 15, 16].

The European Food Safety Authority (EFSA) has conducted comprehensive risk assessments on several phthalates, including DBP, BBP, DEHP, DINP, and DIDP. For DBP, BBP, DEHP, and DINP, a tolerable daily intake (TDI) of 50 μg/kg body weight per day has been established, based on their anti‐androgenic effects, particularly reductions in fetal testosterone levels. In contrast, DIDP, which does not disrupt androgen signaling, has been assigned a higher TDI of 150 μg/kg body weight/day, derived from liver toxicity data [8, 9].

Given the ubiquity of phthalates and their potential health impacts, particularly in the context of PET bottled water, there is a pressing need for improved monitoring, regulation, and consumer awareness. Enhanced quality control, clearer labelling, and greater support for safe tap water alternatives could contribute significantly to reducing population‐level exposure to these persistent and potentially harmful substances. The phthalate concentration of PET bottled mineral water may vary with pH, storage time, storage temperature, and exposure to sunlight. Photolysis may be a significant pathway for abiotic degradation of phthalates in waters [12, 17, 18, 19, 20, 21].

Given the widespread detection of phthalates in PET bottled water despite regulatory restrictions, further investigation into the sources, mechanisms, and extent of contamination is warranted.

Several analytical methods have been developed for the determination of phthalates, which generally involve labor‐intensive extraction procedures based on the use of organic solvents. Among these approaches, liquid–liquid extraction (LLE) remains one of the most commonly employed techniques [22, 23, 24]. LLE is considered a reliable and effective method; however, it presents several drawbacks. It requires the use of toxic organic solvents, such as dichloromethane, *n*‐hexane, or acetone, which efficiently extract phthalates from the sample matrix. The excessive use of organic solvents poses significant risks, as these substances are often toxic and hazardous, leading to reduced environmental sustainability. Another widely used approach is solid‐phase extraction (SPE), which relies on the selective retention of analytes on ion‐exchange cartridges or reverse‐phase (RP) sorbent disks, followed by elution with an appropriate organic solvent [23, 24, 25, 26]. While SPE reduces solvent consumption compared to LLE and allows preconcentration of analytes at very low levels, it remains a relatively complex and time‐consuming procedure, involving multiple steps such as sorbent conditioning, sample loading, washing, and elution, which increase operational complexity and analysis time. The various techniques applied for the extraction of phthalates from water samples are summarized in Table 1. In recent years, alternative techniques such as magnetic solid‐phase extraction (MSPE) [27, 28], salt‐assisted liquid–liquid extraction (SALLE) [29], and liquid–liquid microextraction (LLME) [31, 32] have been proposed. Compared to conventional LLE, these methods reduce solvent consumption and enhance extraction efficiency, thereby mitigating environmental impact. However, despite their high sensitivity and widespread application, they still raise sustainability concerns due to the continued use of toxic solvents, significant energy requirements, and the generation of hazardous waste. To overcome these limitations, solid‐phase microextraction (SPME) was selected as a more environmentally friendly and sustainable alternative. SPME is a solvent‐free technique that enables the selective extraction of target analytes directly from the sample matrix with minimal sample handling. This approach reduces the risk of secondary contamination during the pretreatment phase and limits operator exposure to toxic organic solvents. All extracted analytes are then transferred directly to the analytical instrument for subsequent analysis. SPME is a cost‐effective, highly sensitive, and rapid technique [33, 34].

**TABLE 1:** Comparison of different extraction methods for the determination of phthalates in water samples.

Analyte	Extraction method	Analytical method	LOD (μg/L)	LOQ (μg/L)	Recoveries (%)	Organic solvent	References
DEHP	LLE	GC–MS/MS	0.05	0.1	40–100	Dichloromethane, *n*‐hexane	[22]
DBP, DEHP	SPE	GC/MS	0.9, 2.34	1.02, 4.13	90–97	Ethyl acetate, methanol	[25]
DBP, DEHP	SPE	LC/MS	0.29, 0.16	0.97, 0.53	56–125	*n*‐Hexane, 2‐propanol, acetonitrile	[26]
DBP, DEHP	MSPE	HPLC/UV	0.003, 0.016	0.02, 0.055	80–114	*n*‐Hexane, acetone	[27]
DEHP, DBP	MSPE	GC/FID	0.031, 0.017	—	97.5–102	Acetone, methanol, dichloromethane	[28]
16 phthalates	SALLE	GC–MS/MS	001‐2	—	70–118	Acetonitrile	[29]
DBP, DEHP	RDSE	GC/MS	0.01, 0.03	0.1, 0.09	86–79	Ethyl acetate, methanol	[30]
DEP, DBP	TSP‐LLME	GC/MS	0.007, 0.12	0.02, 0.35	82.2–105.6	*n*‐Hexane, acetone, dichloromethane, ethyl acetate	[31]
DBP	DSPE‐DLLME	GC/FID	1.24	4.11	84–91	Acetonitrile, methanol, acetone, 2–propanol, ethyl acetate	[32]
DEHP	Methanol dilution	LC/MS	0.5	1.5	80–120	Methanol	[21]
DBP; DEHP	SPME	GC/MS	0.3, 0.99	0.77, 2.57	92–96	—	[33]

Abbreviations: DBP, di‐*n*‐butyl phthalate; DEHP, di(2‐ethylhexyl) phthalate; DSPE, dispersive solid‐phase extraction; GC–MS/MS, gas chromatography–mass spectrometry/mass spectrometry; GC/FID, gas chromatography–flame ionization detector; GC/MS, gas chromatography–mass spectrometry; HPLC/UV, high‐performance liquid chromatography with ultraviolet detection; LC/MS, liquid chromatography‐mass spectrometry; LLE, liquid–liquid extraction; LLME, liquid–liquid microextraction; MSPE, magnetic solid‐phase extraction; RDSE, rotating disk adsorptive extraction; SALLE, salting out assisted liquid‐liquid extraction; SPE, solid‐phase extraction; SPME, solid‐phase microextraction; TSP, temperature‐sensitive polymer.

This study aims to identify and quantify the presence of phthalates in water in plastic bottles made of both virgin and recycled PET using solid‐phase microextraction coupled with gas chromatography–mass spectrometry (SPME‐GC/MS).

## 2 Materials and Methods

### 2.1 Chemicals and Materials

Bis(2‐ethylhexyl) phthalate (DEHP) and ultrapure water were purchased from Sigma‐Aldrich (Milan, Italy), while dibutyl phthalate (DBP) was purchased from Carlo Erba (Cornaredo, Italy). SPME fibers coated with divinylbenzene/carboxen/polydimethylsiloxane (DVB/CAR/PDMS) were purchased from Supelco (Merck KGaA, Darmstadt, Germany). SPME screw‐top vials (40 mL) with metal caps were obtained from Gerstel (Baltimore, MD, USA).

### 2.2 Samples

Seventeen commercial bottled mineral water samples from various brands, including virgin PET (*n* = 11) and recycled PET (*n* = 6), were acquired from local supermarkets in Palermo (Sicily, Italy). All samples were stored at room temperature and analyzed within their shelf‐life period to reflect typical storage conditions.

### 2.3 Preparation of Standard and Sample Solutions

Individual analytical standards were not available for all the phthalates investigated (see Table 2). Therefore, a relative quantification approach was employed. Diethyl phthalate (DEP) and diisobutyl phthalate (DiBP) were quantified and referred to dibutyl phthalate (DBP). For bis(2‐ethylhexyl) phthalate (DEHP), a standard stock solution was prepared.

**TABLE 2:** PAEs identified in this study and their selected physicochemical properties.

Name	Abbreviation	Molecular weight	Chemical structure	Formula	CAS
Diethyl phthalate	DEP	222.24	C_12_H_14_O_4_	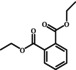	84‐66‐2
Diisobutyl phthalate	DiBP	278.34	C_16_H_22_O_4_	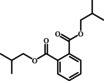	84‐69‐5
Dibutyl phthalate	DBP	278.34	C_16_H_22_O_4_	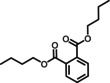	84‐74‐2
Bis(2‐ethylhexyl) phthalate	DEHP	390.56	C_24_H_38_O_4_	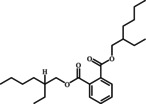	117‐81‐7

Stock solutions of DBP and DEHP were prepared in hexane at concentrations of 1100 and 630 mg/L, respectively. Calibration was performed using external standard curves. For DBP, five calibration levels were prepared in ultrapure water at concentrations of 0.2, 1, 5, 10, and 25 μg/L. For DEHP, calibration solutions were prepared at concentrations of 0.9, 1, 5, 10, and 25 μg/L. Calibration solutions were prepared by diluting individual stock solutions with ultrapure water that had been preanalyzed to confirm the absence of phthalates.

### 2.4 Extraction Procedure of Phthalates and Analysis by SPME‐GC/MS

Solid‐phase microextraction (SPME), a green and environmentally sustainable solvent‐free technique, is widely applied for the determination of trace‐level organic compounds. This technique is based on the adsorption and subsequent desorption of analytes using a fused silica fiber coated with a polymeric stationary phase, enabling the extraction of volatile and semivolatile compounds from gaseous, liquid, or solid matrices [21, 27, 28, 29, 30, 31, 32, 34, 35, 36, 37, 38]. The experimental parameters, such as extraction time, temperature, fiber selection, desorption conditions, and subsequent GC/MS analysis, were optimized through a series of preliminary trials.

For each analysis, 10 mL aliquots of water samples were transferred into 40 mL glass vials sealed with silicone/PTFE septa. Extraction was performed at 25°C for 30 min under continuous magnetic stirring using direct immersion SPME (DI‐SPME) with a triphasic divinylbenzene/carboxen/polydimethylsiloxane fiber (DVB/CAR/PDMS), which is particularly suited for a broad range of analytes including phthalates [34]. DI‐SPME involves directly immersing the fiber into the liquid sample for the duration of extraction.

The water sample was subjected to controlled agitation to enhance mass transfer, thereby improving extraction efficiency and reproducibility. Prior to use, the SPME fiber was conditioned in the GC injector at 270°C for 30 min according to the manufacturer's specifications. After the extraction phase, the fiber was retracted into the hollow needle of the syringe, removed from the vial, manually introduced into the GC injector port and desorbed for 3 min at 260°C [34]. Each sample was analyzed in triplicate to ensure analytical reproducibility.

#### 2.4.1 GC/MS Analysis and Conditions

Analyses were carried out using a Thermo Fisher Scientific TSQ 8000 triple quadrupole mass spectrometer coupled with a Thermo Fisher Scientific Trace 1310 gas chromatograph (Waltham, MA, USA).

Samples were separated using a TG XLBMS column (20 m × 0.18 mm ID × 0.18 μm, Thermo Scientific, GC column). The injector temperature was maintained at 260°C. Helium (99.9995%) was used as the carrier gas at a constant flow rate of 1.0 mL/min. The oven temperature was held at 50°C for 3 min, increased to 300°C at a rate of 40°C/min and held for 10 min. Mass spectrometric detection was performed in positive electron ionization (EI) mode with an ionization energy of 70 eV and a source temperature of 300°C. Mass spectra were acquired in full‐scan mode over the m/z range 35–500. Each analyte was identified by matching the extracted ion chromatogram and retention time with those of the corresponding reference standard, when available (Table 2), and by comparing the mass spectrum against the NIST 2018 library database.

### 2.5 Quality Assurance and Control

The GC/MS method was assessed in terms of repeatability, reproducibility, linearity, limit of detection (LOD), and limit of quantification (LOQ). Repeatability refers to the precision of the method under the same experimental conditions within a short time frame (intraday), while reproducibility describes the variability of the analytical response under the same conditions but across different days or analytical sessions (interday). Precision was expressed as the relative standard deviation (%RSD), and accuracy was evaluated using the percentage relative error (%RE).

LOD and LOQ were determined following the guidelines of the American Chemical Society's Committee on Environmental Analytical Chemistry, based on blank samples treated identically to the water samples. These values were estimated from the calibration curve regression parameters, calculated as: LOD = 3 *× σb/a* and LOQ = 10 *× σb/a* where *σb* is the standard deviation of the blank signal and *a* is the slope of the calibration curve.

Sample concentrations were quantified using calibration curves specific to each analyte; when a reference standard was unavailable, the calibration curve for DBP was used as a reference.

### 2.6 Methodology for Risk Assessment

To evaluate the potential human health risks from phthalate exposure via mineral water, we employed a risk assessment approach comparing the estimated daily intake (DI) with the EFSA tolerable daily intake (TDI), established at 50 μg/kg body weight (bw) per day for both DBP and DEHP [8, 9]. The DI (μg/kg bw/day) for each sample was calculated using the following equation:

(1) $$\mathrm{DI}=\frac{C\times \mathrm{IR}}{\mathrm{Wb}}$$

where *C* is the phthalate concentration in the water sample (μg/L), *IR* is the daily ingestion rate (2 L/day for adults and 1.5 L/day for toddlers), and *Wb* denotes the body weight (60 kg for adults and 12 kg for toddlers) [39, 40].

These values were subsequently compared to the EFSA TDI, and the risk quotient (*RQ*) was calculated as the ratio of DI to TDI, defined by the following equation:

(2) $$\mathrm{RQ}=\frac{\mathrm{DI}}{\mathrm{TDI}}$$

An *RQ* value less than 1 indicates exposure below the threshold of concern, implying no significant health risk.

## 3 Results and Discussion

All 17 water samples were analyzed using SPME in direct immersion mode with a commercially available DVB/CAR/PDMS fiber. This fiber exhibited superior extraction efficiency for the target phthalates, mainly attributable to the Carboxen porous sorbent layer, which is well‐suited for extracting smaller polar molecules [34]. The experimental conditions for the GC/MS analyses were optimized to ensure optimal chromatographic separation and maximum sensitivity. The main advantage of using direct immersion SPME as a sample pretreatment method is its operational simplicity, which greatly lowers the risk of secondary contamination during sample handling. Additionally, direct immersion improves extraction efficiency for polar compounds and reduces the formation of artefacts caused by fiber coating saturation. This technique also requires only small sample volumes, minimizes operator exposure to hazardous organic solvents, and promotes environmentally friendly extraction practices.

To evaluate the performance of the developed method, several validation parameters were assessed, including linearity, LOD, LOQ, intraday and interday precision, and recovery. The calibration curves demonstrated excellent linearity, with correlation coefficients (*R* ^2^) exceeding 0.993.

The LOD was 0.30 μg/L for DBP and 0.77 μg/L for DEHP, while the LOQ was 0.99 μg/L for DBP and 2.57 μg/L for DEHP. The RSD% values for both intraday and interday precision were consistently below 10% for DBP and below 5% for DEHP, thus showing good measurement precision.

Method accuracy was evaluated using fortified samples prepared by spiking blank matrix (bi‐distilled water) with known quantities of DPB and DEHP. The accuracy expressed as relative error (RE%) was 8% for DPB and 6% for DEHP. The calculated analytical parameters confirmed the reliability of the proposed approach. The low values of LOD and LOQ suggest that the proposed technique is suitable for rapid screening of phthalates in mineral water.

Furthermore, consistently higher recovery rates of 92% for DBP and 96% for DEHP, verified by independent analyses of the same mineral water on different days, demonstrate the robustness of the method.

**FIGURE 1:**
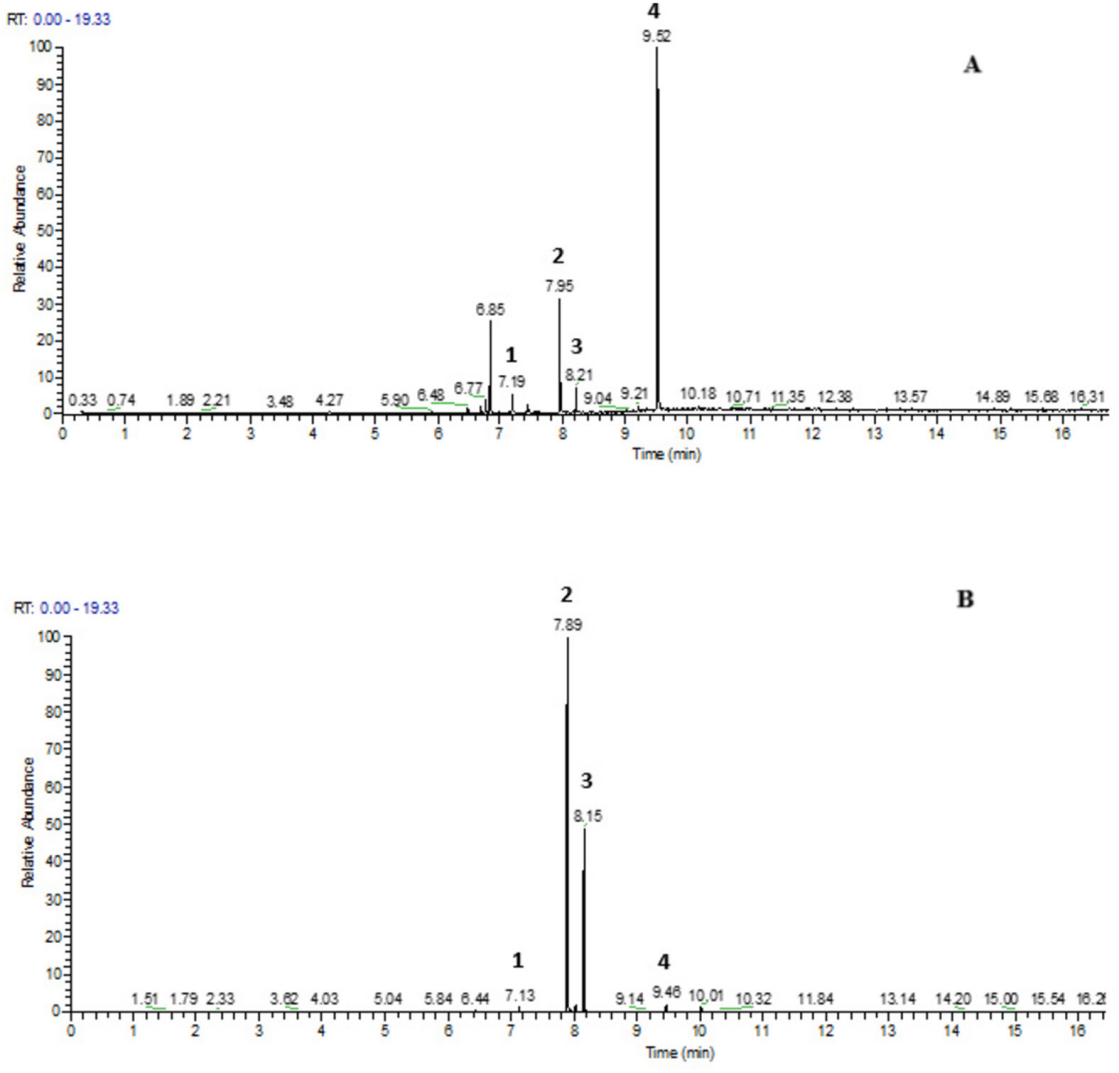
GC/MS chromatograms of water by virgin PET bottle (sample B1) and recycled PET bottle (sample RB5‐50%) [B] showing the extracted ion at m/z 149. The compounds eluted in the following order: **1**, diethyl phthalate, **2**, diisobutyl phthalate, **3**, dibutyl phthalate, and **4**, di(2‐ethylhexyl) phthalate.

**FIGURE 2:**
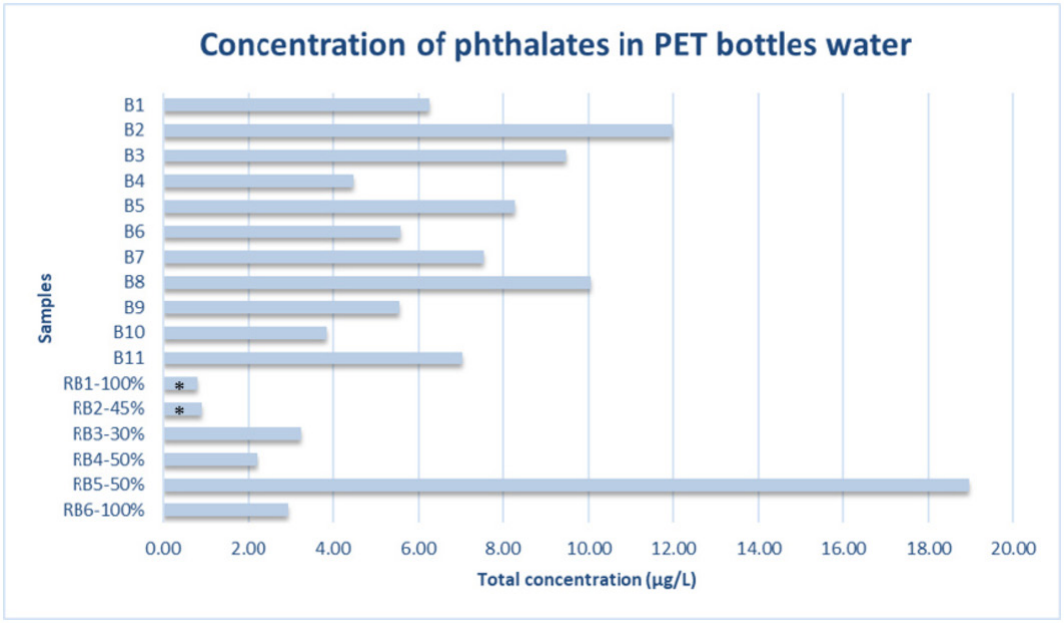
Total concentration of phthalates (μg/L) in mineral water of PET bottles; the * symbol indicates that the values are less than the LOD.

Phthalates identified and quantified in the PET bottled water samples included DEP, DiBP, DBP, and DEHP as shown in the chromatograms (Figure 1). As shown in Table 3, the concentrations of these four phthalates detected in 17 water samples from commercial virgin PET bottled water (B1–B11) and recycled PET bottled water (RB1–RB6) with varying recycling content (30%–100%) are reported. Total phthalate concentrations in these samples ranged from 2.21 to 18.59 μg/L. The distribution of total phthalate concentrations across all samples is presented in Figure 2.

**TABLE 3:** Phthalate concentrations (μg/L) in commercial virgin PET bottled water (B) and recycled PET bottled water (RB‐%); the percentage indicated in RB samples refers to the proportion of recycled PET used in the bottle composition.

No.	Sample	DEP	DiBP	DBP	DEHP	Total
1	B1	< LOD	< LOD	< LOD	6.27 ± 0.05	6.27 ± 0.05
2	B2	< LOD	< LOD	< LOD	11.97 ± 0.06	11.97 ± 0.06
3	B3	< LOD	< LOD	< LOD	9.49 ± 0.05	9.49 ± 0.05
4	B4	< LOD	< LOD	< LOD	4.49 ± 0.56	4.49 ± 0.56
5	B5	< LOD	5.31 ± 0.01	1.80 ± 0.01	1.16 ± 0.01	8.28 ± 0.01
6	B6	< LOD	3.60 ± 0.01	0.87 ± 0.01	1.21 ± 0.02	5.57 ± 0.02
7	B7	< LOD	5.09 ± 0.02	1.60 ± 0.06	0.86 ± 0.01	7.54 ± 0.06
8	B8	< LOD	5.33 ± 0.02	2.11 ± 0.02	2.60 ± 0.02	10.04 ± 0.02
9	B9	< LOD	2.43 ± 0.01	0.85 ± 0.01	2.27 ± 0.01	5.55 ± 0.01
10	B10	< LOD	1.32 ± 0.02	0.64 ± 0.13	1.89 ± 0.01	3.85 ± 0.13
11	B11	0.70 ± 0.01	3.73 ± 0.02	0.99 ± 0.13	1.62 ± 0.01	7.05 ± 0.01
12	RB1‐100%	< LOD	< LOD	< LOD	< LOD	< LOD
13	RB2‐45%	< LOD	< LOD	< LOD	< LOD	< LOD
14	RB3‐30%	< LOD	1.96 ± 0.01	0.42 ± 0.01	0.88 ± 0.01	3.26 ± 0.01
15	RB4‐50%	< LOD	0.35 ± 0.01	< LOD	1.86 ± 0.66	2.21 ± 0.66
16	RB5‐50%	< LOD	10.01 ± 2.63	4.60 ± 1.50	4.35 ± 2.53	18.96 ± 2.63
17	RB6‐100%	< LOD	1.40 ± 0.005	0.37 ± 0.01	1.19 ± 0.01	2.95 ± 0.01

*Note:* The results indicate the mean values ± SD (standard deviation) of the counts performed in triplicate.

All four phthalates were detected only in the B11 sample, DEP was not detected (< LOD) in any of the samples except for this sample, with a concentration of 0.70 μg/L, while DEHP was the most prevalent phthalate, detected in all 17 samples.

The highest phthalate concentration was observed for DEHP in sample B2 (11.97 μg/L), followed by DiBP in sample RB550% (10.01 μg/L) and DEHP in B3 (9.49 μg/L).

Total phthalate concentrations in water from virgin PET bottle samples ranged from 3.85 to 11.97 μg/L, with a median of approximately 7 μg/L.

The sample RB1‐100% and RB2‐45% showed all values < LOD for individual analytes. The other samples of water from recycled PET bottles (RB3‐30%–RB6‐100%) showed the presence of multiple phthalates, with significantly elevated levels in the RB5‐50% sample, where DiBP reached 10.01 μg/L and total phthalates exceeded 18 μg/L. The elevated phthalate concentration observed in sample RB5‐50%, relative to the other recycled PET samples, may be attributed to intrinsic contamination of the recycled feedstock, inadequate decontamination during processing, or external sources such as environmental contamination. This result underscores the necessity of robust quality control protocols throughout the food‐grade PET recycling process. Such oversight is particularly critical given the growing emphasis on plastic reuse and integration into circular economy frameworks, where the potential for contaminant carryover can represent a significant challenge to both product safety and regulatory compliance.

Total phthalate concentrations in water from recycled PET bottle samples ranged from 2.21 to 18.96 μg/L, with a median of approximately 6.84 μg/L.

While water from virgin PET bottles tended to exhibit consistently higher total phthalate concentrations, water from recycled PET bottles was characterized by greater variability and generally lower concentrations. Except for sample RB5‐50%, total phthalate concentrations detected in water from recycled PET bottles were very low.

### 3.1 Evaluation of Potential Human Health Risks

To assess the potential human health risk associated with phthalate exposure through mineral water, the EFSA's TDI values [8, 9] were used as reference points. Using the measured phthalate concentrations, the estimated DI and RQ were calculated for each water sample, and the results are summarized in Table 4. None of the analyzed samples exceeded the TDI limits.

**TABLE 4:** Estimated daily intakes (DIs) and risk quotient (RQ) for adults (body weight of 60 kg) and toddlers (body weight of 12 kg) considering a daily water consumption of 2 L for adults and 1.5 L for toddlers in PET bottles of mineral water.

	DI adults		DI toddlers		RQ adults		RQ toolers	
Sample	DBP (μg/kg bw)	DEHP (μg/kg bw)	DBP (μg/kg bw)	DEHP (μg/kg bw)	DBP	DEHP	DBP	DEHP
B1	—	0.21	—	0.78	—	4.20 × 10^−3^	—	1.57 × 10^−2^
B2	—	0.40	—	1.50	—	8.0 × 10^−3^	—	3.0 × 10^−2^
B3	—	0.32	—	1.19	—	6.32 × 10^−3^	—	2.40 × 10^−2^
B4	—	0.15	—	0.56	—	2.30 × 10^−3^	—	1.12 × 10^−2^
B5	0.06	0.04	0.23	0.14	1.20 × 10^−3^	7.73 × 10^−4^	4.51 × 10^−3^	2.90 × 10^−3^
B6	0.03	0.04	0.10	0.15	5.11 × 10^−4^	8.06 × 10^−4^	1.92 × 10^−3^	3.02 × 10^−3^
B7	0.05	0.03	0.20	0.11	1.07 × 10^−3^	5.72 × 10^−4^	4.00 × 10^−3^	2.14 × 10^−3^
B8	0.07	0.09	0.26	0.33	1.41 × 10^−3^	1.73 × 10^−3^	5.29 × 10^−3^	6.50 × 10^−3^
B9	0.03	0.08	0.11	0.28	5.69 × 10^−4^	1.51 × 10^−3^	2.14 × 10^−3^	5.67 × 10^−3^
B10	0.02	0.06	0.08	0.24	4.24 × 10^−4^	1.26 × 10^−3^	1.59 × 10^−3^	4.73 × 10^−3^
B11	0.03	0.05	0.12	0.20	6.57 × 10^−4^	1.08 × 10^−3^	2.47 × 10^−3^	4.05 × 10^−3^
RB1‐100%	—	—	—	—	—	—	—	—
RB2‐45%	—	—	—	—	—	—	—	—
RB3‐30%	0.01	0.03	0.05	0.11	2.78 × 10^−4^	5.85 × 10^−4^	1.04 × 10^−3^	2.19 × 10^−3^
RB4‐50%	—	0.06	—	0.23	—	1.24 × 10^−3^	—	4.66 × 10^−3^
RB5‐50%	0.15	0.14	0.58	0.54	3.07 × 10^−3^	2.90 × 10^−3^	1.15 × 10^−2^	1.09 × 10^−2^
RB6‐100%	0.01	0.04	0.05	0.15	2.45 × 10^−4^	7.94 × 10^−4^	9.17 × 10^−4^	2.98 × 10^−3^

In particular, the concentrations of DBP and DEHP, both identified by the EFSA as substances of concern due to their adverse effects on reproductive health, were well below their respective TDI thresholds [8]. Because all RQ values were below 1, indicating exposure levels under the threshold of concern, these findings suggest that, under the conditions tested, phthalate presence in mineral water stored in PET bottles does not pose a significant health risk for either adults or toddlers.

## 4 Conclusions

This study highlights the effectiveness and reliability of a sustainable and efficient SPME‐GC/MS method for the detection and quantification of selected phthalates in water samples stored in both virgin and recycled PET bottles. The analytical method demonstrated high sensitivity, precision, and accuracy, along with low detection and quantification limits, making it well‐suited for routine monitoring applications.

All samples analyzed showed estimated DIs and the RQ of all detected phthalates below the limits set by EFSA, both for adults and infants, indicating that there is no immediate risk to human health through the consumption of PET bottled water. However, a notable variability in phthalate content was observed, particularly in recycled PET bottles (RB‐%). While it is important to note that most RB samples had lower concentrations of phthalates than virgin bottles, only one sample (RB5‐50%) showed significantly elevated levels, suggesting possible contamination from the recycling process or previous use of the plastic. This highlights the importance of strict quality control and standardized certification procedures for recycled PET intended for food‐contact use. Overall, the results support the viability of recycled PET in water packaging, provided that proper decontamination and regulatory oversight are in place. The validated method offers a practical tool for ongoing surveillance and risk assessment of phthalate migration in bottled PET water.

## Funding

This work was supported by the University of Palermo (Budget 10% dottorato 2025).

## Data Availability

The data that support the findings of this study are available from the corresponding author upon reasonable request.
